# Which triggers could support timely identification of primary antibody deficiency? A qualitative study using the patient perspective

**DOI:** 10.1186/s13023-021-01918-x

**Published:** 2021-06-29

**Authors:** Lisanne M. A. Janssen, Kim van den Akker, Mohamed A. Boussihmad, Esther de Vries

**Affiliations:** 1 Department of Tranzo, TSB, Tilburg University, PO Box 90153, 5000 LE Tilburg, The Netherlands; 2 Department of Pediatrics, Amalia Children’s Hospital, Nijmegen, The Netherlands; 3 Radboud University, Nijmegen, The Netherlands; 4 Jeroen Bosch Academy Research, Jeroen Bosch Hospital, ’s-Hertogenbosch, The Netherlands

**Keywords:** Primary antibody deficiency, Qualitative research, Timely diagnosis, Diagnostic journey, Patient perspectives, Trigger

## Abstract

**Background:**

Patients with predominantly (primary) antibody deficiencies (PADs) commonly develop recurrent respiratory infections which can lead to bronchiectasis, long-term morbidity and increased mortality. Recognizing symptoms and making a diagnosis is vital to enable timely treatment. Studies on disease presentation have mainly been conducted using medical files rather than direct contact with PAD patients. Our study aims to analyze how patients appraised their symptoms and which factors were involved in a decision to seek medical care.

**Methods:**

14 PAD-patients (11 women; median 44, range 16-68 years) were analyzed using semi-structured interviews until saturation of key emergent themes was achieved.

**Results:**

Being always ill featured in all participant stories. Often from childhood onwards periods of illness were felt to be too numerous, too bad, too long-lasting, or antibiotics were always needed to get better. Recurrent or persistent respiratory infections were the main triggers for patients to seek care. All participants developed an extreme fatigue, described as a feeling of physical and mental exhaustion and thus an extreme burden on daily life that was not solved by taking rest. Despite this, participants tended to normalize their symptoms and carry on with usual activities. Non-immunologists, as well as patients, misattributed the presenting signs and symptoms to common, self-limiting illnesses or other ‘innocent’ explanations. Participants in a way understood the long diagnostic delay. They know that the disease is rare and that doctors have to cover a broad medical area. But they were more critical about the way the doctors communicate with them. They feel that doctors often don’t listen very well to their patients. The participants’ symptoms as well as the interpretation of these symptoms by their social environment and doctors had a major emotional impact on the participants and a negative influence on their future perspectives.

**Conclusions:**

To timely identify PAD, ‘pattern recognition’ should not only focus on the medical ‘red flags’, but also on less differentiating symptoms, such as ‘being always ill’ and ‘worn out’ and the way patients cope with these problems. And, most important, making time to really listen to the patient remains the key.

**Supplementary Information:**

The online version contains supplementary material available at 10.1186/s13023-021-01918-x.

## Background

Rare diseases are defined as occurring in less than 1:2000 people. However, since there are around 8000 rare diseases, some 30 million people in both Europe and in the USA suffer from a rare disease. This is an important problem for the patients as well as for the society they live in because rare diseases are often diagnosed late, especially when they share symptoms with common diseases, leading to delayed and inadequate treatment. As a consequence, these patients suffer a decreased life quality as well as a decreased potential for societal participation (school, work) [1, 2].

Predominantly (primary) antibody deficiencies (PADs) are a typical example of such difficult-to-recognize rare diseases [3]. Hypogammaglobulinemias are by far the most common forms of PAD, comprising nearly half of all primary immunodeficiency (PID) diagnoses [4–6]. Affected persons commonly develop recurrent otitis media, sinusitis, and pneumonia. Recurrent pneumonias can lead to bronchiectasis, which serves as a negative factor for long-term morbidity and mortality. Since the introduction of immunoglobulin replacement therapy, there have been dramatic improvements in survival [7, 8]. Recognizing symptoms and making a diagnosis is therefore vital to enable timely treatment.

Although PADs are the most common primary immunodeficiencies (PIDs) in humans, they are still rare with a prevalence of approximately 1:25,000 to 1:110,000, depending on the type of PAD [3]. These patients often go unrecognized, because the general public as well as most healthcare professionals, who are not specialized in immunodeficiency, do not consider PAD in patients with recurrent “normal” infections. Because of the variability of presenting clinical manifestations, patients visit various physicians of different specialties in search of a diagnosis, which increases the risk of missing the overarching clinical pattern and thereby overlooking the underlying hypogammaglobulinemia [9]. Timely diagnosis and treatment will likely result in improved clinical and quality-of-life outcomes for patients with PAD, higher participation in society (school, work) and lower healthcare costs [10–15]. Reducing diagnostic delay is therefore crucial.

Studies on disease presentation have mainly been conducted using medical files rather than direct contact with PAD patients. We aimed to explore the presenting pattern of PAD from the perspective of patients and to identify factors that affect a correct and timely diagnosis, by exploring the period leading to the PAD diagnosis through narrative patient interviews.

## Materials and methods

### Design and setting

Patient experiences regarding their journey to receiving a diagnosis and adequate clinical care are best understood via an in-depth qualitative approach. Through individual semi-structured interviews with patients who had a diagnosis of PAD, new insights derived from their perspective were sought. Their experiences and reasoning regarding complaints and the diagnostic delay they suffered were explored. New interviews were conducted until saturation of key emergent themes was achieved, meaning additional interviews were no longer adding new themes to the data set. Methods and results are reported according to the COREQ checklist [16].

### Population

Participants were recruited by an email sent by the Dutch patient organization for primary immunodeficiency diseases to all their members (‘Stichting voor Afweerstoornissen’, SAS). The email invited the members to participate in an interview of about one hour at a place of their choice and stated that it would be audiotaped. All participants provided written and audio informed consent. The interviews were primarily with study participants but the contribution of a relative, if present, was also welcomed.

### Data collection

The interviews were performed in October and November 2016 at the patients’ homes. The interviews were conducted by a master student in the last year of medical education (KvA). Questions were semi-structured, designed to address those items which the interviewer wished to raise, and also allowing participants the freedom to express their own perspective and to offer an opportunity for serendipitous findings. The questions (overview in Additional file 1) were based on the literature related to clinical characteristics of primary antibody deficiency [9, 17–19] and to psychosocial theories relating to symptom appraisal and care-seeking [20]. All interviews were reviewed and discussed with an experienced medical immunologist with expertise in qualitative methods (EdV).

### Data analysis

All interviews were audiotaped and literally transcribed (KvA and MAB), and analyzed using the framework method [21]. Data were anonymized by removing any information that could identify the patient. Transcripts were read and re-read to ensure familiarization, and independently coded (MAB and LJ). The coding was reviewed by a third coder (EdV), to ensure that the type and range of codes applied was appropriate and consistent. The coding lists were used to develop a framework organized into categories. In total, we identified 154 codes we divided into 11 categories, which were organized in 3 themes (Additional file 2). The coding was finalized using the software package Atlas.ti Version 7.1.5 (Berlin, Germany), and the coded data were exported (MAB). This export was read, re-read and then summarized for each of the 14 participants of the study. Each category was then interpreted using an analytical memo to explore emerging themes and concepts.

## Results

In total, 14 participants were interviewed. Interviews lasted from 45 to 105 min. Eleven women and three men participated, median age 44 years, range 16–68; all participants were Dutch. Participant characteristics are summarized in Table 1. Three interviewees were accompanied by a relative during the interview. The results are presented under subheadings reflecting the main steps in the diagnostic pathway, namely: presentation of PAD and participants’ interpretation of symptoms, progression of symptoms and realization that something is really wrong, starting the patient journey, doctors’ interpretation of symptoms, and triggers to diagnosis. Participants were also asked to reflect on care provision and on the emotional toll of the diagnostic process.

**Table 1:** Symptom attribution and delay before diagnosis

Patient	Age (years)			Delay (years)	Signs and symptoms	Patient’s attribution
	At start of symptoms	At time of diagnosis	At time of interview			
1, F, CVID	28	33	38	5	Recurrent sinusitis/ otitis/ rhinitis/ pneumonia, fatigue, weight loss, anosmia, splenomegaly	Increased susceptibility due to pregnancy, being too busy and taking too little rest
2, F, sIgAdef	13	32	35	19	Chronic rhinitis, hypothyroidism, fatigue, stomach and bowel complaints	No considerations, but fear about the diagnosis
3, F, CVID	26	29	35	3	Chronic cough, recurrent otitis/ bronchitis, ITP, alopecia areata, chronic fatigue	Some kind of autoimmune disorder, sensitive lungs
4, F, CVID	43	46	51	3	Being always ill, almost continuously fever, recurrent rhinitis/ otitis/ pneumonia/ sinusitis, anosmia, fatigue, recurrent ITP, chronic diarrhea, meningitis, inguinal lymphadenopathy, weight loss	n/a
5, M, agammaglobulinemia	4	13	59	9	Recurrent meningitis/ pneumonia/ otitis/ sinusitis	XLA (after diagnosed was discovered in his brother)
6, F, CVID	45	51	57	6	Recurrent respiratory infections/ sinusitis/ pneumonia, chronic cough, aphthous lesions, salpingitis, arthralgia, bronchial hyperreactivity, fatigue, exercise intolerance	Some kind of immune disorder
7, F, unPAD	0	5	36	5	Recurrent otitis/ rhinitis/ sinusitis, chronic cough, skin abscess, pneumonia, failure to thrive	n/a
8, M, CVID	5	40	58	35	Recurrent otitis/ rhinitis/ sinusitis/ pneumonia/ varicella zoster/ Giardia lamblia, fatigue, warts, meningitis, anosmia	n/a
9, F, unPAD	22	42	46	20	Recurrent otitis/ sinusitis/ pneumonia/ skin infections, mumps, chickenpox (2x), asthma, Graves’ disease	Initially Graves’ disease and asthma, later after searching the internet an immune disorder
10, F, CVID	8	23	24	16	Erythema nodosum, splenomegaly, enlarged supraclavicular lymph node, fatigue, oral aphthous lesions, being always ill, recurrent otitis/ sinusitis	Iron deficiency anemia, some kind of viral infection
11, M, sIgAdef	0	4	16	4	Recurrent rhinitis/ otitis/ pharyngitis, fatigue, growth retardation, chronic diarrhea	n/a
12, F, IgG subclass deficiency	1	40	44	39	Recurrent sinusitis/ pharyngitis/ respiratory tract infections, fatigue, multiple allergies, asthma, retropharyngeal abscess	Combination of (severe) asthma and allergies
13, F, Good syndrome	Not precisely known	68	68	> 30	Iron deficiency anemia, recurrent lymphadenopathy/ cystitis/ sinusitis/ otitis/ respiratory tract infections, fatigue, chronic diarrhea, diverticulitis	Combination of iron deficiency anemia, asthma and diverticulitis
14, F, CVID	40	50	63	10	Recurrent sinusitis and pneumonia, odontogenic infections, sepsis, severe wound infection, fatigue, exercise intolerance, chronic slightly elevated body temperature	Initially viral infections in combination with psychological factors (divorce) and menopause, later after searching the internet an immune disorder

* CVID* common variable immunodeficiency disorders, *F* female, *IgGscdef* IgG subclass deficiency, *ITP* idiopathic thrombocytopenic purpura, *M* male, *n/a* not applicable, *PID* primary immunodeficiency, *slgAdef* selective IgA deficiency, *unPAD* unclassified primary antibody deficiency, *XLA* X-linked agammaglobulinemia

### Presentation of primary antibody deficiency and participants’ interpretation of symptoms

The presenting features of PAD described by participants were diverse, intermittent and sometimes non-specific, covering a broad range of behavioral and physical changes (Table 1). Being always ill featured in all participant stories. It often occurred from childhood onwards and was considered to be a problem by participants and/or their parents when periods of illness were felt to be too numerous, too bad, too long-lasting, or when antibiotics were always needed to get better.

Then I got my penicillin course, then it was over within two days, but then the penicillin course was over again and two days later it started all over again. (Participant 4)

Many participants thought recurrent infections to be normal for children, or in some cases due to atopic disease. Seven adult participants just felt ill and did not have a particular explanation for why they were more often ill than others but assumed that the symptoms would probably resolve with time. Participants tended to downplay and/or normalize their symptoms.

When it started with asthma, I had two to four respiratory tract infections a year. That’s not very strange, it is not what strikes you as abnormal if you have asthma. (Participant 9)

I think everybody coughs sometimes with a little mucus, but then I don’t feel very sick. Usually, it resolves with time. Eventually, when I was diagnosed with CVID, they did a Chest-CT. Then they saw the beginning damage that fits the clinical picture and lungs. They also said: ‘how is that possible, you said you only had pneumonia once, but from looking at the scan it seems as if it really cannot have been just once’. (Participant 10)

Fatigue was present both before and after the diagnosis in many participants and was described as a feeling of physical and mental exhaustion and thus an extreme burden on daily life. Most participants recalled that they slept very well, but still remained tired. The decrease in energy level could result in the need for an afternoon nap.

And really a lot of fatigue, I slept for three hours during the day and ten hours at night. I really slept thirteen to fifteen hours a day. Just to keep up. (Participant 1)

I spoke about it with my sister recently. I always thought, everybody works the whole day, five days a week, but I actually can’t do that. It would tire me so badly, it’s not possible. (Participant 6)

Participants described they thought they exerted themselves too much, causing their symptoms themselves by working too hard, taking care of their whole family or by being too active socially. The degree of fatigue could be significant before the participant really labelled it as a problem. Eleven participants reported their fatigue was so severe it took away all their free time, because after a work/school day there was no energy left for social activities.

Then I sleep the whole night, being just able to fulfil the expectations set during the day. I don’t have any free time left, because when I come home, I go to sleep, and then another day begins. (Participant 10)

Participants had comorbidities, complications or misdiagnoses, and tended to attribute their symptoms to these conditions. For example, a participant with iron deficiency anemia attributed her fatigue solely to this condition, and a participant with Graves’ disease attributed her fatigue solely to that. Whereas in both participants these conditions could well be complications of their—unrecognized—PAD. Another participant with a misdiagnosis of asthma thought her fatigue was the result of needing more potent inhalers. In total, four participants (1, 7, 9 and 12) attributed their symptoms to asthma.

At that time I got diagnosed with asthma, for which I had to use inhalers that just did not work. (Participant 7)

Participants carried on with their usual activities despite significant limiting symptoms.

Just tired… I always went to work anyway, …what good is it to you to lie down on the couch all day. (Participant 4)

I have always done fitness at a fairly high level and I did that three times a week. At one point it became less and less due to fatigue. That I just couldn’t manage to exercise for an hour at eight o’clock in the evening. Sometimes I had to force myself to do it, but then I just took an extra puff, so I could do it again. (Participant 1)

Often, unusual and alarming signs for PID were not recognized as unusual medical conditions by the consulting doctors, for example: ITP, alopecia areata and recurrent infections in participant 3; recurrent chicken pox in participants 8 and 9; excessive oral aphthous lesions in participant 10; recurrent meningitis in participant 5; recurrent otitis in adult patients repeatedly needing tympanostomy tubes in participants 1, 2 and 8; excessive weight loss in participants 1 and 4; salpingitis after swimming in participant 6, and impaired wound healing in participant 9.

### Progression of symptoms and realization that something is really wrong

Typically, the symptomatology evolved over weeks to months, with non-specific early features such as recurrent “normal” infections, fever, chronic cough and fatigue, mimicking those of common, self-limiting illnesses. Some participants described a triggering event (thyroid disease, pregnancy, weight loss or severe wound infection after caesarean section) as starting point for a sudden increase in infections. Symptoms often progressed over time. Infections slowly became abnormally recurrent, severe and/or persistent.

It starts with only periods of coughing and then at one point it’s actually all the time. (Participant 3)

Four participants (4, 6, 8 and 12) suffered from chronic rhinosinusitis and underwent sinus surgery. This resulted in only a short relief of complaints. In the sons of participant 8, multiple sinus surgeries were performed in addition to weekly nasal irrigation and polypectomy. The ENT specialist was alarmed by their voluminous medical file.

But the ENT-specialist told us: ‘you are right, at the whole ENT-department we have nobody with a file as large as those of your sons’. We came there for only three years. He said: ‘those files are now already bigger than the files of a fifty-year old’. (Participant 8)

Nine participants (1, 3–6, 8, 9, 12 and 14) recalled their infections only resolved with antibiotic treatment.

As soon as I got my antibiotics intravenously in the hospital, I recovered. So everyone was like, you’re fine again, you can go home. So yeah, it was okay for a while, only after four episodes of six days, so four weeks of illness, they wanted to conduct further research. (Participant 1)

Participants often recalled a pattern in the complaints. In participants 1 and 12 the upper respiratory tract infection (otitis, pharyngitis and/or sinusitis) always progressed to a lower respiratory tract infection.

It progressed from an infection of the sinuses, to the ears and then to the throat and airways. (Participant 1)

It often began with infections: lungs, sinus, yes very often my sinuses, and then it spread to my lungs and throat at the same time. (Participant 12)

Four participants (1, 2, 3 and 8) repeatedly developed otitis—often after swimming—in adulthood and were treated with tympanostomy tubes.

Well, I mean, everybody might have an ear infection once a while, but every time I had it, it lasted a month. That I really had so much pain for a month, that you just wanted to hit your head against the wall, because you don’t know what to do about the pain anymore. Then the doctor told me: ‘yes, but antibiotics do not help against an ear infection, so I do not really want to give that’. Then it lasted just really long every time, but the GP did not think: ‘oh, that is weird’. (Participant 3)

Participants 7 and 9 realized they differed from other people when comparing the duration of recovery.

I often had a cold. Another person had it for two days and if I had it was for two months. (Participant 7)

I played handball and that is not a ‘sweet’ sport, to be fair, my wounds recovered badly… That was very weird, the wound always got infected or it took four weeks to heal. With the other kids in my environment the wounds always recovered within a week or two. For me never, it always took longer. (Participant 9)

Participant 6 recalled her infections to start rapidly and become severe in a short time.

It could be that one moment I thought: ‘I’m going to make it’, but then an hour later I would be so ill that I didn’t make it. (Participant 6)

The burden of infections was perceived to be susceptible to change. Participants pointed out that they experienced positive as well as negative fluctuations in the burden of infections due to weather conditions: four patients reported being sick throughout the year (1, 2, 4, 8); three patients were almost never ill during the summer (5, 6, 9).

All except two participants were working age and initially thought that their symptoms were a normal part of their busy lifestyle and job, but once recognized as abnormal by others (employers, colleagues) they sought help. Family members often witnessed participants struggling with symptoms and encouraged them to seek help. Five participants (1, 2, 4, 5 and 7) often recalled that their social environment thought they were sicker than they admitted themselves.

I just went to my job, I have often been sent home by my boss. (Participant 1)

That’s also what my colleagues said, how often I was at the office with a sinusitis or otitis, that everybody was like: ‘you shouldn’t do that, you’re ill’. Yes, I am very often ill, this isn’t even that bad. (Participant 1)

Most participants were quick to seek medical advice from their GP as soon as they realized something was really wrong. However, it took most participants a long time to realize this; they continued to hope that the symptoms would simply disappear.

### Starting the patient journey

The factors which triggered the seeking of care were various. One patient sought help because of the psychological stress she suffered due to the many unexplained symptoms. Recurrent or persistent infectious episodes like recurrent otitis, sinusitis, or pneumonia or endless coughing, were the main triggers for patients to seek care. Often the fatigue and strain of coping in life while hindered by all the symptoms were the reason to go—again—to the GP. Combinations of problems could also be the final push.

Participants reported cumulative barriers that led to delays to seek help. Their interpretation of the initial signs and symptoms of the disease influenced whether they sought help. Participants either got used to their symptoms or hoped their symptoms would pass by.

At a certain point you raise the bar and think by yourself: ‘I am not using medicines or visit the doctor again’. You think: ‘I just keep taking pills’. Then you raise the bar again and wait and see for another day. (Participant 1)

Well, in the beginning you go to the doctor… But when you are always ill, you just don’t go to the doctor anymore. (Participant 3)

Others no longer sought help because the healthcare professional only treated symptoms instead of searching for the cause. Before the CVID diagnosis was made in patient 8, he underwent recurrent sinus surgeries resulting in only short relief of the (chronic) sinusitis. These experiences kept him from seeking help after a while. Another theme that emerged was feeling delegitimized. This led them to feel distressed by the way they were treated, by not being believed or listened to and not being able to cope with symptoms. Participants reported the feeling that both healthcare professionals and others did not see them as having a legitimate illness and that the credibility of their symptoms was frequently questioned. Patient 3 reported that her symptoms were for a long time attributed to an unacknowledged mental health condition and which made her feel that the symptoms were not due to an underlying pathology.

I went to the doctor, who thought: ‘I think it’s not that bad how often you are sick’. So then she said: ‘I think it is how you experience it, that it is in your head, but not real’. So then she initially referred me to a psychologist. (Participant 3)

Over time, having healthcare professionals questioning their credibility made participants question the legitimacy of their symptoms too. Participants described feeling guilty for wasting healthcare professionals’ time, or downgrading their symptoms by normalization, waiting till another infectious episode passed by.

Once patients began to seek a diagnosis, delays also occurred within the healthcare system. Clinicians were not often familiar with PID and were challenged by the complexity and rarity of the disease. This impacted their ability to make a differential diagnosis. Sometimes healthcare professionals seemed to have difficulties in abandoning an initial diagnosis. Participant 6 was treated for bacterial pneumonia, but her general state of health worsened with loss of condition, leading to the inability to climb the stairs. She suggested herself to screen for possible PID because of recurrent Hemophilus Influenzae pneumonia despite adequate antibiotic treatment, after which CVID was discovered. In addition to physician inflexibility, this case reveals the importance of communication concerning symptoms from everyday life as well as medical symptoms. Six participants (1, 6, 8, 9, 10 and 14) recalled their social environment forming a barrier in seeking help. The social environment of these participants downplayed the participants’ complaints. They attributed the symptoms to stress/a busy life or told the participants it would resolve with time.

‘It is probably because of the stress’, people say that a lot too. (Participant 10)

### Doctors’ interpretation of symptoms

Many participants recalled initially being offered an incorrect explanation for their symptoms (Table 2). Most doctors initially attributed the participant’s symptoms to minor, viral illnesses, asthma, anatomical ENT-problems or to other ‘innocent’ explanations such as ascribing joint pains to sporting activities, episodic dyspnea to stress-induced hyperventilation, feeling worn out to the combination of working too hard and taking care of a newborn child, erythema nodosum to mosquito bumps and exercise intolerance to menopause. In one participant XLA, despite a positive family history, was only discovered years after he already had several episodes of meningitis. In one participant, her symptoms were put down to being pregnant.

**Table 2:** The journey towards a diagnosis of primary antibody deficiency

Patient	The diagnostic pathway						
1	Doctor	GP	ENT specialist (1st trajectory)	ENT specialist (2nd trajectory)	Pulmonologist	Oncologist	
	Signs and symptoms	Recurrent upper airway infections	Recurrent upper airway infections	Recurrent upper airway infections	Chronic cough, episodic dyspnea, especially at night	Recurrent upper airway infections, weight loss, frequent hospital admission for respiratory infections, night sweats, splenomegaly	
	Attribution	n/a	Nasal polyps	n/a	Asthma	Leukemia, non-Hodgkin lymphoma	
	Action	Referral to ENT specialist	Polypectomy	Prednisone, antibiotics, tympanoplasty	Pulmonary function test, increasing the dose of inhalation corticosteroids, prophylactic antibiotics	Hospital admission, extensive examinations leading to CVID diagnosis	
2	Doctor	GP (1st trajectory)	ENT specialist	GP (2nd trajectory)	Gastro-enterologist	Immunologist	
	Signs and symptoms	Chronic rhinitis, chronic fatigue, hypothyroidism	Chronic rhinitis	Stomach and bowel complaints, chronic fatigue, frequent GP visits	Stomach and bowel complaints, infiltrative enterocyte lesions (Marsh 1)		
	Attribution	Chronic rhinitis not further specified	Nasal septum deviation	Gastritis not further specified	Irritable bowel syndrome		
	Action	Referral to ENT specialist	Septoplasty, steroid nasal spray	Antacids, and after persistent symptoms, referral to gastro-enterologist	Gluten-free diet was considered, peppermint oil, referral to immunologist after IgA-deficiency was discovered		
3	Doctor	GP (1st trajectory)	Psychologist	GP (2nd trajectory)	Patient	Immunologist	
	Signs and symptoms	Chronic cough, recurrent otitis, burn-out symptoms	Feeling worn out, burn-out symptoms	Chronic cough, recurrent otitis, burn-out symptoms	Chronic cough, recurrent otitis, burn-out symptoms, ITP, alopecia areata	See under ‘patient’	
	Attribution	Recurrent bronchitis in combination with psychological factors	The combination of being always ill, working and taking care of a newborn child	n/a	Some kind of auto-immune disease	Immunologic or auto-immune disorder	
	Action	Antibiotic treatment, bronchodilators, referral to psychologist	Referral back to GP	Advise to the patient to google to find out the cause of complaints	Arranging own referral to immunologist/ rheumatologist	Extensive laboratory investigations after which the CVID diagnosis was made	
4	Doctor	GP (1st trajectory)	Pulmonologist	ENT specialist	GP (2nd trajectory)	Pulmonologist	Immunologist
	Signs and symptoms	Recurrent rhinitis/ pneumonia/ sinusitis	Recurrent rhinitis/ pneumonia/ sinusitis	Recurrent rhinitis/ pneumonia/ sinusitis	Persistent, recurrent respiratory infections, meningitis	Persistent, recurrent respiratory infections, meningitis	Persistent, recurrent respiratory infections, meningitis, inguinal lymphadenopathy, weight loss
	Attribution	n/a	Obstruction of sinus drainage, bacterial pneumonia	Obstruction of sinus drainage	n/a	Bacterial pneumonia	PID
	Action	Referral to pulmonologist	Chest X-ray, therapeutic and prophylactic antibiotic treatment, referral to ENT specialist	Endoscopic sinus surgery	Referral to pulmonologist	Chest X-ray, antibiotics, referral to immunologist after IgA-deficiency was discovered	Extensive laboratory investigations after which the CVID diagnosis was made
5	Doctor	GP	Immunologist				
	Signs and symptoms	Recurrent meningitis, otitis, chronic sinusitis, positive family history	Recurrent meningitis, otitis, chronic sinusitis, positive family history				
	Attribution		PID				
	Action	Referral to immunologist	Extensive laboratory investigations after which the XLA diagnosis was made				
6	Doctor	GP	Pulmonologist (1st trajectory)	Pulmonologist (2nd trajectory)			
	Signs and symptoms	Recurrent respiratory infections / sinusitis / pneumonia, bronchial hyperreactivity, fatigue, exercise intolerance	Recurrent respiratory infections / sinusitis / pneumonia, bronchial hyperreactivity, fatigue, exercise intolerance	*Streptocococcus pneumoniae* pneumonia and persistent *Haemophilus influenzae* colonization despite antibiotic treatment			
	Attribution	n/a	Bacterial pneumonia and asthma	Possible CVID			
	Action	Referral to pulmonologist	Sputum cultures, therapeutic and prophylactic antibiotic treatment	After discovery of low serum immunoglobulins, treatment with intravenous immunoglobulins			
7	Doctor	Pediatrician					
	Signs and symptoms	Recurrent otitis / rhinitis / sinusitis, chronic cough, skin abscess, pneumonia, failure to thrive					
	Attribution	PID					
	Action	Extensive laboratory investigations after which the unPAD diagnosis was made					
8	Doctor	GP (1st trajectory)	Pulmonologist	ENT specialist	GP (2nd trajectory)	Immunologist	
	Signs and symptoms	Recurrent otitis/ rhinitis/ sinusitis/ pneumonia	Recurrent otitis/ rhinitis/ sinusitis/ pneumonia	Recurrent otitis/ rhinitis/ sinusitis/ pneumonia	His two sons were diagnosed with CVID by a pediatrician	Recurrent otitis/ rhinitis/ sinusitis/ pneumonia, two sons were diagnosed with CVID by a pediatrician, recurrent varicella zoster and Giardia lamblia infections, warts, anosmia	
	Attribution	n/a	Bacterial pneumonia	Nasal septum deviation/ polyps	Possible CVID	Possible CVID	
	Action	Referral to ENT specialist and pulmonologist	Prophylactic and repeated therapeutic antibiotic treatment	Prophylactic and repeated therapeutic antibiotic treatment	Referal to immunologist	Extensive laboratory investigations after which the CVID diagnosis was made	
9	Doctor	GP (1st trajectory)	GP (2nd trajectory)	GP (3rd trajectory)	Pulmonologist	ENT specialist	Immunologist
	Signs and symptoms	Recurrent otitis/ sinusitis/ skin infections, poor wound healing, chicken pox (2x), mumps	Dyspnea, wheezing, chronic cough	Fatigue, stomach and bowel complaints	Dyspnea, wheezing, chronic cough, recurrent respiratory infections	Recurrent sinusitis and pneumonia despite PnPS and Hib vaccination and antibiotic treatment	Recurrent sinusitis and pneumonia despite PnPS and Hib vaccination and antibiotic treatment
	Attribution	Recurrent infections in infancy	Asthma	Graves’ disease	Asthma	Possible PID	Possible PID
	Action	None	Inhalation corticosteroids, referral to pulmonologist	Antithyroid medication	Increasing the dose of inhalation corticosteroids, repeatedly oral prednisolone and antibiotic treatment	Functional endoscopic sinus surgery and referral to immunologist	Extensive laboratory investigations after which the unPAD diagnosis was made
10	Doctor	GP (1st trajectory)	GP (2nd trajectory)	GP (3rd trajectory)	GP (4th trajectory)	Internist (1st trajectory)	Internist (2nd trajectory)
	Signs and symptoms	Fatigue, aphthous lesions	Erythema nodosum	Erythema nodosum + splenomegaly	Erythema nodosum + splenomegaly, enlarged supraclavicular lymph node	Erythema nodosum + splenomegaly, enlarged supraclavicular lymph node	Erythema nodosum + splenomegaly, enlarged supraclavicular lymph node
	Attribution	Iron deficiency anemia	Mosquito bites	Some kind of viral infection	Possible malignancy	Sarcoidosis	
	Action	Iron supplementation	‘Wait and see’	Blood test showed mild pancytopenia; initially ‘wait and see’	Referral to internist	Exclusion of lymphoma after histological examination of lymph node, chest X-ray, discussion in a multidisciplinary team	After suggestion of a colleague to test for immunoglobulins, the diagnosis of CVID was made
11	Doctor	GP	Pediatrician (1st trajectory)	ENT specialist	Pediatrician (2nd trajectory)	Pulmonologist	Immunologist
	Signs and symptoms	Recurrent rhinitis/ otitis/ sinusitis, fatigue, growth retardation, chronic diarrhea	Recurrent rhinitis/ otitis/ sinusitis, fatigue, growth retardation, chronic diarrhea	Recurrent rhinitis/ otitis/ sinusitis, fatigue, growth retardation, chronic diarrhea	Persistent infections despite prophylactic antibiotics and multipe ENT surgeries, extreme fatigue, growth retardation	Persistent infections despite prophylactic antibiotics and multipe ENT surgeries, extreme fatigue, growth retardation	Persistent infections despite prophylactic antibiotics and multipe ENT surgeries, extreme fatigue, growth retardation
	Attribution		Possible celiac disease, recurrent infections in infancy	Reactive mucosa, recurrent infections in infancy	Combination of recurrent infections in infancy and psychological factors	Possible CF/PCD	Possible selective IgA-deficiency
	Action	Referral to ENT specialist en pediatrician	Referral to dietician, prophylactic antibiotics after low IgA was discovered during screening for celiac disease	Tonsillectomy, adenotomy, tympanoplasty, functional endoscopic sinus surgery	Referal to psychologist en pulmonologist	Analyses for CF and PCD were negative; referral to immunologist	Selective IgA-deficiency confirmed
12	Doctor	Pediatrician	Pulmonologist (1st trajectory)	ENT specialist	Pulmonologist (2nd trajectory)		
	Signs and symptoms	Recurrent sinusitis/ pharyngitis/ respiratory tract infections, fatigue	Multiple hospital admisions due to asthma (> 40x)	Recurrent sinusitis/ pharyngitis/ respiratory tract infections, fatigue, retropharyngeal abcess	Still frequent asthma exacerbations despite high-dose inhalation corticosteroids		
	Attribution	(Severe) asthma and multiple allergies	(Severe) asthma and multiple allergies	Reactive mucosa, bacterial infections	(Severe) asthma and multiple allergies		
	Action	Inhalation corticosteroids, referal to pulmonologist and ENT specialist	Frequently oral prednisolone, increasing the dose of inhalation corticosteroids, repeatedly antibiotics, subcutaneous epinephrine always available	Abscess drainage, tonsillectomy, multiple sinus surgeries	IgG-subclass deficiency discovered after immunological screening		
13	Doctor	GP (1st trajectory)	GP (2nd trajectory)	GP (3rd trajectory)	Internist (1st trajectory)	Internist (2nd trajectory)	Pulmonologist
	Signs and symptoms	Iron deficiency anemia, recurrent lymphadenopathy and cystitis, fatigue	Recurrent respiratory infections (including proven pneumonia)/ sinusitis/ otitis	Chronic diarrhea, abdominal pain	Chronic diarrhea, abdominal pain	Persistent abdominal pain, vomiting, recurrent respiratory infections	Persistent abdominal pain, vomiting, recurrent respiratory infections
	Attribution	Some kind of viral infection	Asthma, bacterial pneumonia	Possible diverticulitis	Possible diverticulitis	n/a	CVID, possible bronchiectasis
	Action	Follow-up	Antibiotics, inhalation corticosteroids, oral prednisolone	Referal to internist	Abdominal CT confirmed diverticulitis and kidney stones	Extensive laboratory investigations after which CVID was diagnosed, referal to pulmonologist for screening for bronchiectasis	Thymoma was coincidentally found on chest CT scan, Good syndrome was diagnosed
14	Doctor	GP (1st trajectory)	Gynaecologist	GP (2nd trajectory)	Psychiatrist	Patient	Immunologist
	Signs and symptoms	Recurrent sinusitis and pneumonia, odontogenic infections, sepsis	Severe wound infection after cesarean section	Fatigue, exercise intolerance	Fatigue, exercise intolerance	Recurrent sinusitis and pneumonia, odontogenic infections, sepsis, fatigue, exercise intolerance, persistent *Helicobacter Pylori* and *Giardia lamblia* infections despite treatment, recurrent cystitis, chronic slightly elevated body temperature	Recurrent sinusitis and pneumonia, odontogenic infections, sepsis, fatigue, exercise intolerance, persistent *Helicobacter Pylori* and *Giardia lamblia* infections despite treatment, recurrent cystitis, chronic slightly elevated body temperature
	Attribution	Viral and bacterial infections	Bacterial infection	Menopause and psychological factors	Psychological factors	Possible PID	Possible PID
	Action	Repeatedly antibiotics	Prophylactic antibiotics during second cesarean section	Referral to psychiatrist	Treatment for stress (not further specified)	Arranging own referral to immunologist	Extensive laboratory investigations after which CVID was diagnosed

*CF* cystic fibrosis, *CVID* common variable immunodeficiency disorders, *ENT* ear-nose-throat, *F* female, *IgGscdef* IgG-subclass deficiency, *ITP* idiopathic thrombocytopenic purpura, *GP* general practitioner, *M* male, *n/a* not applicable, *PCD* primary ciliary dyskinesia, *PID* primary immunodeficiency, *slgAdef* selective IgA-deficiency, *unPAD* unclassified primary antibody deficiency, *XLA* X-linked agammaglobulinemia

Participants were referred to several different specialists (Table 2), often only after strongly insisting on it. In two participants, their symptom attribution to psychological factors, led to multiple visits to a psychiatrist.

I did not have severe infections, but I couldn’t do anything anymore. Well, what happens then: ‘psychic, menopause, divorce’. I started believing that after a while. Then I went to a psychiatrist. I went there for years. (Participant 14)

Two participants (2 and 8) appeared to have one or more decreased immunoglobulin isotypes years before the final diagnosis, but this was not noticed by the doctor or the doctor did not know what this meant and ignored the results. This reflects the problem that most non-immunologists have minimal or no knowledge of PID. Even treatment failure—implying an unusual disease course—did not alarm an ENT specialist to think of potential PID.

I had meningitis in 2011 and then I recovered and they thought it went better, but then I became sick again. Then I went to the ENT-doctor… They cleaned my sinuses. That was February 2012. Then I became sick again, they didn’t understand it anymore. Then I ended up in the hospital again in April. (Participant 4)

### Triggers to diagnosis

In none of the participants, the symptoms were attributed to potential PID by the GP, except for one participant, in whom his children were already diagnosed with PID. Only two participants were referred directly to an immunologist, where referral immediately led to a correct diagnosis. Two participants were diagnosed through a positive family history, although one of them had already suffered a meningitis eight times (which had not triggered the potential diagnosis). In two participants (2 and 11) PID was incidentally discovered while screening for celiac disease (low serum IgA).

Multidisciplinary consultations can support the diagnostic process. In participant 10, who had splenomegaly, erythema nodosum and pancytopenia, one of the specialists recognized the symptom pattern and suggested to test for immunoglobulins.

Eventually the internist told me: ‘I actually don’t know what you have, I think it’s sarcoidosis, but your blood doesn’t show that’… ‘I’m going to discuss you one more time in a multidisciplinary consultation, if we don’t find it then, then we really don’t know’. In that consultation I think one smartass said: ‘test for antibodies’. (Participant 10)

Alarm symptoms can trigger the diagnosis. For example, healthcare professionals were triggered to conduct additional investigations when participant 1 and 4 suffered from excessive weight loss. Sometimes PID was diagnosed while searching for another diagnosis. Participant 1 suffered from weight loss, night sweats and splenomegaly and was screened for leukemia or lymphoma. Instead, she was found to have CVID. Participant 3 suffered from idiopathic thrombocytopenia and alopecia and was screened for some form of autoimmune disease. Her IgG was found to be decreased instead of elevated; she was diagnosed with CVID.

They started searching for an autoimmune disease and they determined the total serum IgG level. They expected that to be super high, because they thought of lupus or something like that. But it was very low at that time. (Participant 3)

Abnormal symptom patterns can trigger the healthcare professional to conduct further investigations. In participant 9 the ENT specialist noticed inflammation on the inside of the nose, usually indicating allergic rhinitis. However, allergy tests were negative and antihistamines, nasal corticosteroids and turbinate reduction did not alleviate her symptoms. This triggered the ENT specialist to refer to an immunologist.

When I entered, he inspected my nose and said: ‘this is what an allergic nose looks like’. I said to him: ‘you can say that, but nobody can prove that I have allergies’. Then they cut it out and cleaned it, but I kept having a lot of complaints. Then he started to look further. (participant 9)

The pediatric PID patients had a shorter diagnostic delay than the adult PID patients in our study. Participant 7 suffered from recurrent otitis, rhinitis and sinusitis, as well as chronic cough, skin abscesses, pneumonia and failure to thrive since birth and was diagnosed with hypogammaglobulinemia at the age of 5 (during follow-up a diagnosis of unclassified primary antibody deficiency was made). The sons of participant 8 were diagnosed with CVID by their pediatrician, triggered by recurrent need for tympanoplasty and sinus surgery. While their father had severe and recurrent infections for several years, he was only diagnosed with CVID after this was discovered in his two children.

Participant 9 and 14 played a direct role in obtaining their PID diagnosis by searching for a cause of their symptoms on the internet and diagnosing a potential PID by themselves.

Participants in a way understand the long diagnostic delay. They know the disease is rare and a GP has to cover a broad medical area. They realize the diagnostic delay is due to not knowing, instead of not wanting to know.

It is of course so rare. There are so many things that doctors should be aware of that I understand that it has not been directly diagnosed. (Patient 1)

I don’t blame her, because you can’t know everything, but I thought: ‘Gosh, I felt really miserable then’. (Patient 2)

But participants are more critical about the way the doctors communicate with them. They feel that doctors often don’t listen very well to their patients. They want the doctor to acknowledge they don’t know what is the matter and to refer the participant to a specialist, and to observe existing guidelines.

The stupid thing is that there is a pulmonology guideline for recurrent airway infections. According to the protocol he had to make a referral. He didn’t know that but then at least be honest enough to say that you don’t know. (Participant 9)

### Emotional toll of the diagnostic delay

During the interviews it became increasingly clear that the participants’ symptoms as well as the interpretation of these symptoms by their social environment and doctors had a major emotional impact. Finally knowing they have probably had PAD for years without being diagnosed also negatively impacted their lives. Participants recalled feeling a lack of understanding by their social environment, who often questioned or downgraded their complaints.

Well I didn’t think it was hard to accept the fatigue and just go to bed. It was often more the incomprehension of others, like: ‘Gosh, already? We just started’. (Participant 1)

Then they think: ‘there she comes again, she is sick again’. (Participant 4)

Eight participants stated that their symptoms had a negative influence on their future perspectives. Struggling with their untreated symptoms they made different choices in their career paths or were hampered in getting promoted.

It always got in the way of everything. Because he was really good at his job, he would be promoted, but then he was ill again. So another person got the job. That was always very depressing. (Wife of participant 8)

In others, symptoms prevented them from doing what made them happy, because they felt themselves to be a burden for their social environment.

Concerts, yes I love that. I didn’t do that in a long time, because it is the worst annoyance of every orchestra member when somebody is coughing in the hall. (Participant 6)

I haven’t, for example, been on vacation for years. I very often felt that I was a burden for other people. When we went on vacation and I was sick again, that’s not what you want. (Participant 6)

Many participants reported that fatigue negatively influenced multiple aspects of their social network. They often could not participate in social activities due to fatigue or had to deal with the consequences of taking part in social activities by sleeping all next day. This led to social isolation and feelings of loneliness.

Every time they say: ‘we can’t come’. At seven o’clock they lie in bed and their friends go out. So then you won’t be asked anymore. (Mother of the sons of participant 8, who also have CVID)

You always have to disappoint people because you have to drop out last-minute. That’s why a lot of CVID patients get isolated, because at some point, you don’t want to disappoint people anymore. (participant 8)

The daily limitations due to the PAD-related complaints were a heavy mental burden for many participants. Eight patients mentioned that they had, to some degree, lost the joy in life.

Of course when being younger I really felt alone, sometimes even depressed. (Participant 12)

## Discussion

This study reveals presenting patterns that can help to identify those patients who are ‘always ill’ and ‘worn out’ *with* PAD. This is important, because recurrent respiratory infections and fatigue are much more prevalent *without* concomitant PAD [22]. Participants in this study tended to normalize their symptoms and carry on with usual activities. Coping strategies of the extreme fatigue that PAD patients develop therefore differ from those with chronic fatigue syndrome, because patients with chronic fatigue syndrome often use escape/avoidance strategies [23]. Also, PAD patients reported to sleep well, whereas chronic fatigue patients generally report more difficulty falling asleep and interrupted sleep [24]. In addition to these non-medical aspects, participants recalled many medical aspects that could trigger suspicion for potential PAD: infections being unusually frequent and/or severe, not clearly season-bound, requiring antibiotics to clear. Many participants underwent repeated sinus surgery, tympanoplasty and/or polypectomy, at best only temporarily resulting in relieve of symptoms. These aspects are in fact already known, but their relevance is often missed in everyday practice. Therefore, to timely identify PAD, ‘pattern recognition’ should not only focus on the medical ‘red flags’, but also on less differentiating symptoms, such as ‘being always ill’ and ‘worn out’ and the way patients cope with these problems. And, most important, making time to really listen to the patient remains the key.

A wide range of factors affected the speed and accuracy of diagnosing PAD. First and foremost, both patients and non-immunologist healthcare professionals tended to persist in misattributing the presenting signs and symptoms to common, self-limiting illnesses or other ‘innocent’ explanations. Both patients and non-immunologist healthcare professionals initially attributed “being always ill” and “feeling worn out” to ‘doing too much’ or ‘sleeping too little’, or to medical conditions such as nasal polyps, nasal septum deviation, asthma or chronic rhinitis. The prevalence of most presenting symptoms of PAD in the general population is high. A population prevalence of 11% is for instance reported for sinusitis, and in a symptom prevalence study 28% of patients experienced coughing in the previous 7 days, and 11% had experienced fatigue [25, 26]. This can explain why ‘normalizing’ prevalent symptoms such as fatigue or recurrent infections is so widespread. The normalization of symptoms and symptom misattribution to less serious or pre-existing conditions have been reported to account for appraisal delays in various cancers, particularly when the early symptoms were commonly occurring non-specific symptoms (e.g., fatigue) [27, 28]. Remarkably, also the appraisal of less common and more alarming signs, such as immune thrombocytopenic purpura (ITP), excessive oral aphthous lesions, recurrent meningitis, and recurrent otitis repeatedly needing tympanostomy tubes in an adult, did not alert both patients and health care professionals to potential underlying disease. Participants were referred to multiple physicians before a diagnosis was made, intensifying the time of worrying and wondering. These findings highlight an important knowledge gap among general practitioners and non-immunologist hospital doctors regarding the clinical presentation of PAD. Education campaigns that address this issue could reduce the time between the onset of symptoms and treatment. In addition, multidisciplinary consultations (MDC’s) with a number of specialists working together can support the diagnostic process for patients presenting with non-specific symptoms who have visited multiple physicians [29]. It would be interesting to explore health care professionals’ views about PAD and about recognizing rare disease. This would help to reveal what health care professionals need to be able to use this knowledge in their own practice.

A second major theme was an increasing reluctance to seek care, albeit in some participants more than others. Reasons for this were diverse. They included getting so used to symptoms that they were considered to be ‘normal’, the feeling of not being taken seriously, and opposing ‘just being treated for symptoms anyway’ instead of being investigated for their cause. The quality of the doctor-patient relationship had a significant impact on the process of obtaining a correct diagnosis. A GP’s prior view of a patient as being ‘thin-skinned’ or ‘a worrier’ could influence how seriously they investigated their complaints. These two themes highlight the full range of factors potentially influencing a timely diagnosis, rather than the presenting medical features of the underlying disease alone.

All this led to a significant mental burden for the participants. The period prior to the diagnosis was particularly challenging, with people feeling dismissed by health care professionals, in spite of their distress. The lack of a definitive diagnosis not only left them open to self-doubt, but also to the negative judgements of others including family, friends and employers. This concept is not unique to PAD as other conditions which are challenging to diagnose, such as systemic lupus erythematosus (SLE), elicit similar ‘journeys’. In patients with SLE, the perception of being dismissed and of the lacking of empathy from a health care professional has been described as leading to feelings of emotional neglect [30]. Participants in this study reported relief when they found a name for their symptoms after they had spent years fighting searching for that. Although the PAD diagnosis helped them by legitimizing their symptoms, validating their suffering and improving their emotional status by being believed and listened to, they still had to cope with the burden of their disease and its treatment for the rest of their lives. While it has already been shown that PAD can result in a considerable disease burden [14, 31], our qualitative study adds the perspective of the patients themselves. Participants found the impact of being ‘always ill’ and ‘worn out’ on their daily functioning to be a key factor determining their emotional well-being. They experienced serious limitations in their social functioning, causing social isolation and feelings of loneliness. This is in line with results from a previous study on COPD-related fatigue, which stated that fatigue influences physical, cognitive, and psychological functioning [32]. Another study related fatigue to perceived health and concluded higher fatigue correlated to a lower perceived health [33]. Other pathologies, such as different types of cancer, showed fatigue being a driver in the impact of quality of life in patients [34–36]. These results highlight the need for more attention to the potential patient burden in the diagnostic delay of PADs.

### Limitations

Because of the relatively small sample, whilst the size of the sample is in keeping with the in-depth nature of qualitative research, this explorative study should perhaps be validated by future studies. The sample included patients who had been diagnosed some years previously. This may have contributed to recall bias. Nevertheless, the study produced a rich amount of material and the findings provide insight into areas of potential future research.

## Conclusions

This study revealed non-medical patterns that can help to recognize patients with potential PAD. With in-depth interviewing, it became clear that—although fatigue can be one of their major complaints—these patients are different from patients with chronic fatigue syndrome: patients with PAD tend to normalize their symptoms and carry on with usual activities. The difficulty experienced by clinicians, as well as patients, in recognizing unusual and alarming signs and attributing symptoms correctly, illustrates that non-immunologists have little knowledge of PAD. This is not surprising, since PAD is a rare disease. Although this underlines the importance of education programs, which should not only focus on the medical ‘red flags’ of PID, but also on coping strategies of more common, less differentiating symptoms, such as ‘being always ill’ and ‘worn out’, this cannot be the final solution. It is impossible for non-experts to know about all > 8000 rare diseases. Hopefully, modern developments in automated pattern recognition can be developed to offer ‘red flags’ in the electronic patient file that alert a physician to potential underlying problems. The results obtained in this study can support the design of predictive models in this regard.

## Supplementary Information

**Additional file 1.** Interview questions.

**Additional file 2.** Codes, categories, and themes used in the qualitative analysis.

## Data Availability

The dataset used and/or analyzed during the current study are available from the corresponding author upon reasonable request.

## Abbreviations

COREQ: consolidated criteria for reporting qualitative research
COPD: chronic obstructive pulmonary disease
CVID: common variable immunodeficiency disorders
GP: general practitioner
ITP: idiopathic thrombocytopenic purpura
PAD: predominantly (primary) antibody deficiency
PID: primary immunodeficiency
SAS: stichting voor afweerstoornissen (Dutch patient organization for immunodeficiency)
