# Supplementary material for: Which triggers could support timely identification of primary antibody deficiency? A qualitative study using the patient perspective
Source: Orphanet J Rare Dis. 2021 Jun 29;16:289. doi: 10.1186/s13023-021-01918-x (PMC8243743; doi:10.1186/s13023-021-01918-x)
Supplement: Supplementary file 1 — Additional file 1. Interview questions. [file 13023_2021_1918_MOESM1_ESM.pdf]

Additional file 1. Interview questions.

<u>Patient characteristics</u>

General characteristics about you [your child].

- Current age: … years.
- Age at which the first symptoms started: … years.
- Age at which the diagnosis ‘deficiency of antibodies’ was made: … years.
- Which diagnosis do you [your child] have?
- Which treatment do you / does your child receive?
  - Immunoglobulins?
  - Antibiotic prophylaxis?

If the interviewee is not the patient himself;

- What is your relationship to the patient?
  - Father / Mother

<u>Introduction to the patient</u>

I would like to hear about the time when you [your child] already had complaints, but the diagnosis ‘deficiency of antibodies’ had not yet been made.

- How long did that period last?
  - When did the complaints start?
  - When was the diagnosis definitely made (patient age)?
- Did the symptoms start suddenly or did they develop gradually?
- What were the complaints during this period?

Together we can try to sketch a short life course. Later, we will discuss the exact complaints and what they meant for you [your child].

- Infant (birth – 18 months)
- Toddler (18 months – 3 years)
- Child (3 years – 5 years)
- Elementary school (6 years – 12 years)
- High school; adolescence (12 years – 18 years)
- Twenties, thirties, et cetera

<u>Medical history</u>

General and central nervous system

- Can you tell me more about your [your child’s] development?
  - Was the children’s healthcare center satisfied about your [your child’s] development?
  - Were you [your child] able to keep up with peers?
- Was there any weight change (decrease or increase)?
- Did you [your child] often have a fever?
  - If so, how often?
  - Was there a pattern in this?
- Do you [your child] have problems with hearing and/or seeing? Or any other problems with the senses (taste, smell, touch)?
- Did you [your child] have a headache, dizziness?
- Are there any memory problems?

Cardiorespiratory

- Did you [your child] have respiratory complaints?
- How is your [your child’s] exercise tolerance?
- Did you [your child] feel fatigued?
  - If so, did this have an influence on daily activities?

Digestive system

- Did you [your child] have a stomach ache?

- Were there any nutritional problems at the time?
- What was your [your child's] stool pattern?

Urogenital
- Did you [your child] have complaints when urinating?

Allergies
- Do you [your child] have allergies?
    - If so, what are you [your child] allergic to?
    - Which symptoms do you [your child] experience after contact with these allergens?
    - Is this allergy confirmed with allergy diagnostics, such as blood- or skin prick test?

Medication
- Did you [your child] use any medicines in the period before the diagnosis 'deficiency of antibodies' was made?
    - If so, for what did you [your child] use these medicines?
    - Who prescribed these medicines?
    - How long did you [your child] take these medicines?
    - Did these medicines have (the desired) effect?
- Did you [your child] use any over-the-counter medications (for example, acetaminophen, aspirin, ibuprofen, oral contraceptive pills)?

Intoxication
- Do you [your child] consume alcohol?
    - How much?
- Do you [your child] smoke?
    - How many cigarettes a day? Since when?
- Do you [your child] use drugs?

Family history
- Are there first- and/or second degree family members with similar complaints?
- Have any first- and/or second degree family members been diagnosed with an immune disorder?
- Have any family members died (because of infections)? If children died at young age, was the cause of death known?
- Is there consanguinity?

Medical history
- Have you [your child] been diagnosed with other illnesses?
- Are you [your child] being treated by a specialist in hospital?
- Have there been repeated hospitalizations?

<u>Non-medical history</u>
Can you tell me more about the family in which you [your child] grew up?
- Parents?
- Brothers and/or sisters?
- Special circumstances?
- Living situation?

Can you tell me more about your [your child's] school-time?
- Which school did you [your child] complete (elementary school, high school)?
- Did you [your child] receive any education?
    - If so, which education?
    - How did this go?
    - Did you [your child] experience any problems?

Can you tell me more about your further adult life?

- Which career choices did you make?
- Did the complaints have consequences for the choices you made?
  - How, what would you have done differently if you had been free of these complaints?
  - Can you tell me more about that?

Can you tell me more about your [your child's] leisure activities?
- Do you [your child] practice a sport?
  - If so, which sport?
  - Performances?
- What are your [your child's] hobbies?

Can you tell me more about your [your child's] social life?
- Do you [your child] have friends?
- Do you [your child] ever go out?
- Did your [your child's] complaints entail restrictions?
- Do you [your child] have enough energy to undertake activities?
