# Supplementary material for: Which triggers could support timely identification of primary antibody deficiency? A qualitative study using the patient perspective
Source: Orphanet J Rare Dis. 2021 Jun 29;16:289. doi: 10.1186/s13023-021-01918-x (PMC8243743; doi:10.1186/s13023-021-01918-x)
Supplement: Supplementary file 2 — Additional file 2. Codes, categories, and themes used in the qualitative analysis. [file 13023_2021_1918_MOESM2_ESM.pdf]

Additional file 2. Codes, categories and themes used in the qualitative analysis.

Theme	Category	Code
**PRE-DIAGNOSIS: JOURNEY TO REFERRAL**	**Self-reported presenting symptoms**	-(Recurrent) fever<br>-Abdominal cramps<br>-Abnormal stool pattern<br>-Arthralgia<br>-Back complaints<br>-Chronically slightly elevated body temperature<br>-Cough<br>-Development of speech<br>-Diarrhea<br>-Dyspnea<br>-Dizziness<br>-Exercise intolerance<br>-Fatigue<br>-Feeling ill<br>-Forgetful<br>-Growth problem<br>-Hair loss<br>-Headache<br>-Hospital admission<br>-Impaired sense of smell<br>-Impaired sense of taste<br>-Lymphadenopathy<br>-Muscle pains<br>-Nasal polyps<br>-Nausea<br>-Night sweats<br>-Overall malaise<br>-Pain<br>-Palpitations<br>-Reduced vision<br>-Stomach and bowel complaints<br>-Swallowing<br>-Tonsillectomy<br>-Tympanostomy<br>-Vomiting<br>-Weight gain<br>-Weight loss
	**Self-reported presenting clinical manifestations**	-Abscesses<br>-Alopecia areata<br>-Allergic reaction to influenza vaccination<br>-Allergic reaction(s)<br>-Aphthous lesions<br>-Aphthous stomatitis<br>-Anemia<br>-Arthrosis<br>-Asthma<br>-Bronchitis<br>-Bronchiectasis<br>-Cataract<br>-Chronic hives<br>-Chronic otitis<br>-Chronic sinusitis

		-Chronic dermatitis<br>-Contact dermatitis<br>-Eczema<br>-Erythema nodosum<br>-Food hypersensitivity<br>-'Flu'<br>-Gastritis<br>-GLILD<br>-Graves' disease<br>-Growth retardation<br>-Headaches/migraine<br>-Hepatitis<br>-Hip injury<br>-Hypertension<br>-Hypothyroidism<br>-Iron deficiency<br>-ITP<br>-'Kind of flu that does not really break through'<br>-Laryngitis<br>-Mucoepidermoid carcinoma<br>-Non-responder to hepatitis A/B vaccination<br>-Peritonitis<br>-Pharyngitis<br>-Pneumonia<br>-Pyelonephritis<br>-Recurrent typical childhood diseases<br>-Recurrent cystitis<br>-Recurrent lower respiratory tract infections<br>-Recurrent otitis<br>-Recurrent meningitis<br>-Recurrent rhinitis<br>-Recurrent sinusitis<br>-Recurrent pharyngitis/tonsillitis<br>-Respiratory tract infections (not further specified)<br>-Salpingitis<br>-Sclerosis<br>-Splenomegaly<br>-Urticaria<br>-Warts
	**Time pattern in clinical manifestations**	-Impact of seasons<br>-Pattern of signs and symptoms<br>-'Life line' of signs and symptoms
	**Symptom appraisal**	-Interpretation of signs and symptoms by patients<br>-Interpretation of signs and symptoms by the social environment<br>-Interpretation of signs and symptoms by the consulting doctor<br>-'Doctors don't know'
	**Emotional toll of the diagnostic process**	-Impact of symptoms on future perspectives<br>-Impact of symptoms on mental well being<br>-Impact of symptoms on relationships<br>-Impact of symptoms on quality of life<br>-Impact of symptoms on regular activities<br>-Battle for legitimacy<br>-The time it took to receive a correct diagnosis<br>-Losing confidence in the healthcare system
	**Coping with symptoms**	-Fighting against symptoms

		-Trivializing symptoms<br>-Adjustments in daily life<br>-Role of the social environment
	**Family history**	-Family member with the same diagnosis<br>-Family member with symptoms suggesting primary immunodeficiency, but not investigated<br>-Family member passed away at young age<br>-Family member passed away because of infections
**DIAGNOSIS: EXPERIENCE OF RECEIVING THE DIAGNOSIS**	**Care-seeking**	-The cue to seek help<br>-The cue for the doctor to conduct further investigations<br>-The patient journey<br>-Fighting for right referrals and pre-diagnosis treatment<br>-Shared decision-making regarding diagnostic analysis / referral<br>-Person who made/suggested the diagnosis<br>-The way the PAD diagnosis was communicated by doctors<br>-Reaction to receiving a PAD diagnosis<br>-Explanation of the diagnosis by the patient<br>-Patient's view on reducing diagnostic delay
**POST-DIAGNOSIS: IMPACT OF THE DIAGNOSIS**	**Issues relating to care-provision**	-Care after the PAD diagnosis<br>-The speed of initial treatment<br>-Shared decision-making regarding treatment<br>-Perceived effect of treatment by patient<br>-Patient autonomy<br>-Patient expertise<br>-Retrospective patient view on care-provision<br>-Disagreement between different doctors<br>-Knowledge about PAD by doctors
	**Impact of the diagnosis and/or treatment**	-Impact of illness and/or treatment on patient's own and family's activities<br>-Impact of illness on being able to work<br>-Post-diagnosis stress / frustrations<br>-Treatment burden<br>-Complications discovered after diagnosis
	**Coping with the diagnosis**	-Adjustments in daily life<br>-Support from the social environment<br>-Developing resilience
